# Soft magnetic properties of nanocrystalline Fe_73_B_7_Si_16_Nb_3_Cu_1_ alloy after rapid heating under tensile stress

**DOI:** 10.1186/s11671-015-0837-z

**Published:** 2015-03-19

**Authors:** Anton Nosenko, Taras Mika, Olexandr Rudenko, Yevhenii Yarmoshchuk, Viktor Nosenko

**Affiliations:** G.V. Kurdyumov Institute for Metal Physics of National Academy of Sciences of Ukraine, 36, Academician Vernadsky Boulevard, Kyiv, 03142 Ukraine; Department of Physics, Taras Shevchenko National University of Kyiv, 64 Volodymyrska Str., Kyiv, 01601 Ukraine

**Keywords:** Amorphous and nanocrystalline Fe-based alloys, Rapid heating by electric current, Tensile stress, Magnetic anisotropy, Magnetic permeability, Core loss

## Abstract

Amorphous Fe_73_B_7_Si_16_Nb_3_Cu_1_ ribbon was crystallized rapidly by electric current heating under simultaneously applied tensile stress along the ribbon axis. As a result, strong transverse magnetic anisotropy was induced in the ribbon. Dynamic magnetic properties of the ribbons rapidly heated either under the tensile stress or without tensile stress were measured using toroidal cores. Optimal electric current heating regime that provides maximum improvement of the initial magnetic permeability and core loss was determined. Tensile stress increase from 0 to 180 MPa was shown to result in the decrease of the initial magnetic permeability down to 400 and core loss at frequencies from 0.4 to 200 kHz. Comparative analysis of magnetic properties of the cut core (with non-magnetic gap) and the cores heated under tensile stress was carried out. The magnetic properties of the latter cores are advantageous for manufacturing the reactors and linear chokes of switch-mode power supplies.

## Background

At present, soft magnetic FINEMET trademark nanocrystalline alloys [1] are widely used in magnetic cores of various inductive components (transformers and chokes). It is known [1] that formation of α-Fe(Si) nanocrystals in FINEMET alloys during annealing improves their soft magnetic properties. Nanocrystalline volume fraction in these alloys is about 80%, and their size is approximately 10 to 12 nm [1-5]. In these alloys and similar ones, a hysteresis loop shape can be changed by inducing uniaxial magnetic anisotropy by annealing in magnetic field [6-8] and/or heating under tensile stress [9-12].

Despite the achievements described, the influence of rapid heating on magnetic properties of FINEMET-type alloys remains insufficiently investigated. In particular, previous studies were focused on the influence of rapid heating under tensile stress on the magnetic properties of amorphous ribbon [13-15]. To the best of our knowledge, there are no studies that demonstrate the advantages of the cores made of the obtained ribbons as compared to the cut (with non-magnetic gap) cores made of the same alloy; the authors of the papers [13,14,16,17] carry out the comparison with the cores with approximately the same permeability but made of other materials. These works show that the cores with induced anisotropy have a number of advantages, the main ones being high-frequency stability of magnetic permeability [13,17], high stability of magnetic permeability, and loss to DC bias field [17] as well as low core loss in the most widely used frequency region (10 to 100 kHz) [13,14,16,17]. Undoubtedly, these advantages attract significant interest to the conditions and possibilities of inducing transverse magnetic anisotropy.

The aims of the present work were to investigate the influence of rapid heating and rapid heating under tensile stress on the magnetic properties of Fe_73_B_7_Si_16_Nb_3_Cu_1_ alloy, to investigate the magnetic properties of cores made of a ribbon heated under tensile stress and cut cores made of Fe_73_B_7_Si_16_Nb_3_Cu_1_ alloy crystallized by conventional isothermal annealing, and to determine the main advantages of the gapless magnetic cores with induced strong transverse magnetic anisotropy.

## Methods

Fe_73_B_7_Si_16_Nb_3_Cu_1_ alloy was obtained in the form of a ribbon with the thickness 20 μm, width 10 mm by planar flow casting process [18]. The planar flow casting process was used for fabrication of the ribbon that was ejected after melting in a quartz tube under CO_2_-protective atmosphere, as described in [19]. The ejection was carried out through the quartz nozzle with rectangular slit (0.4 × 10 mm^2^) at the distance of 200 μm over the wheel (620 mm in diameter, made of chromium bronze). The excessive melt ejection pressure was 20 kPa, the linear rotation speed was 25 m/s, and the melt temperature was 1,400°С.

Straight pieces of the ribbon as well as the ribbon wound into toroidal cores were annealed to obtain nanocrystalline structure. We have determined [4] that the annealing in a furnace for 1.5 h at temperature 550°C in Не atmosphere results in the best soft magnetic properties of these cores.

Rapid heating by AC electric current (20 to 48 A/mm^2^) lasted for 2 to 120 s; this procedure provided the heating of the ribbon above 600°С. After the heating, the ribbon was wound into a core with the inner/outer diameter ratio 9/12.

**Figure 1:**
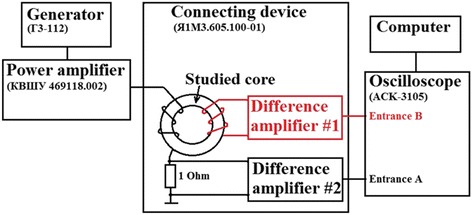
**Principal scheme of MS-02 B-H ANALYZER measuring complex.**

Dynamic B-H curves and core loss at different frequencies were measured using MS-02 B-H ANALYZER measuring complex (MSTATOR, Novgorodskaya oblast, Russia); the functional scheme of this complex is shown in Figure 1. AC current proportional to voltage on the instrument shunt 1 Ohm is applied to magnetizing coil from broadband power amplifier. The measuring device has two precise differential amplifiers no.1 and no.2 which, correspondingly, amplify voltage signal from measuring coil and voltage signal from instrument shunt that is connected in series in the magnetization circuit. Signals from differential amplifiers are registered at inputs B and A, respectively, of virtual digital two-channel storage oscilloscope ASK-3105 installed in the PC system unit. Measurements of remagnetization loop parameters were performed at sinusoidal signal waveform of measuring coil (B channel) at the frequencies from 0.4 to 400 kHz. Measuring system simultaneously with oscillography of dynamic remagnetization loop allowed measuring the values of magnetic field and magnetic induction with the accuracy of ±0.01 А/m and ±0.01 Т, respectively.

Ribbon resistivity was measured by standard four-probe method. X-ray diffraction investigations were carried out by DRON-3.0 M diffractometer (Bourevestnik, St. Petersburg, Russia) using monochromated Мо-K_α_ radiation. X-ray diffraction investigations have shown that the as-quenched ribbon has amorphous structure. Dimensions of nanocrystals were calculated by Selyakov-Scherrer equation [20].

Initial magnetic permeability of magnetic cores was calculated by values of inductance of a few-turn coil in AC field 0.2 А/m at different frequencies measured by LCR Measurement Bridge HM8118 (HAMEG Instruments, Mainhausen, Germany).

## Results and discussion

### Rapid heating of the ribbon by electric current without applying tensile stress

**Figure 2:**
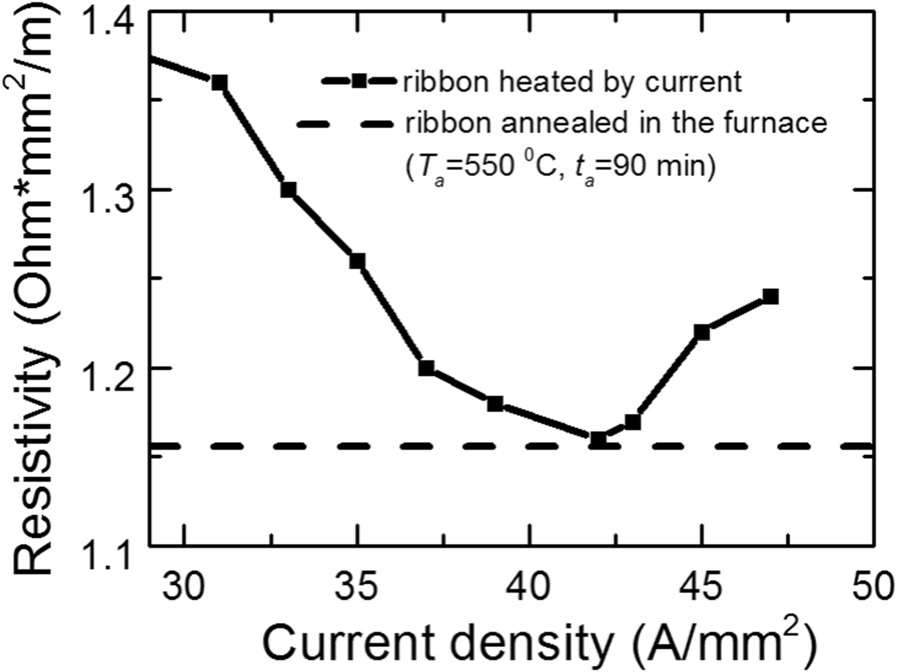
**Ribbon resistivity vs. current density** ***j***_***h***_ **.** Heating time *t*_*h*_ = 10 s.

Figure 2 shows the dependence of ribbon resistivity on current density. Based on this dependence, there is the optimum current density *j*_*h*_ that ensures minimal resistivity *ρ* after heating. Such current provides optimal temperature of the ribbon and its nanocrystallization during the chosen time - 10 s. It is possible that optimal density should be slightly larger for shorter times of heating.

**Figure 3:**
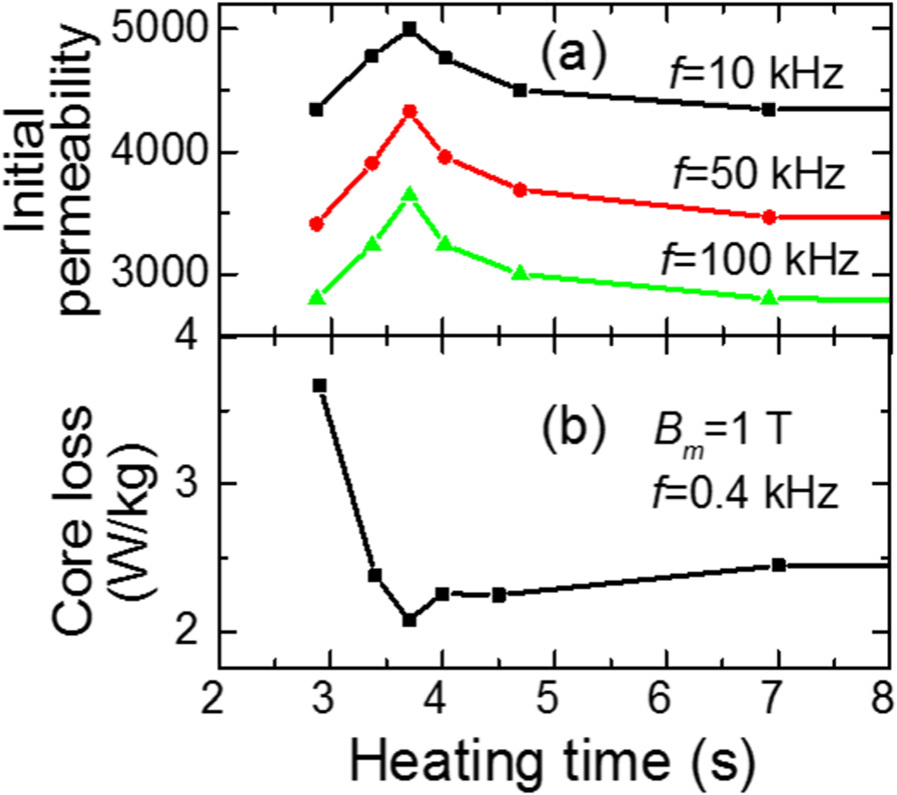
**Dependencies of magnetic properties on heating time.** Current density *j*_*h*_ = 42 А/mm^2^. **(a)** Initial magnetic permeability *μ*_і_ vs. heating time *t*_*h*_. **(b)** Core loss vs. heating time.

Investigations of initial magnetic permeability of the ribbon wounded cores vs. time of ribbon heating by electric current of optimal density (*j*_*h*_ = 42 A/mm^2^) show the existence of optimal time *t*_*h*_ = 3.7 s when the maximum value of initial magnetic permeability *μ*_і_ at different frequencies (10, 50, 100 kHz) (Figure 3a) and minimum power loss per unit mass (hereafter - core loss) at frequency *f* = 0.4 kHz; maximum magnetic induction *B*_*m*_ = 1.0 Т (Figure 3b) is achieved.

**Figure 4:**
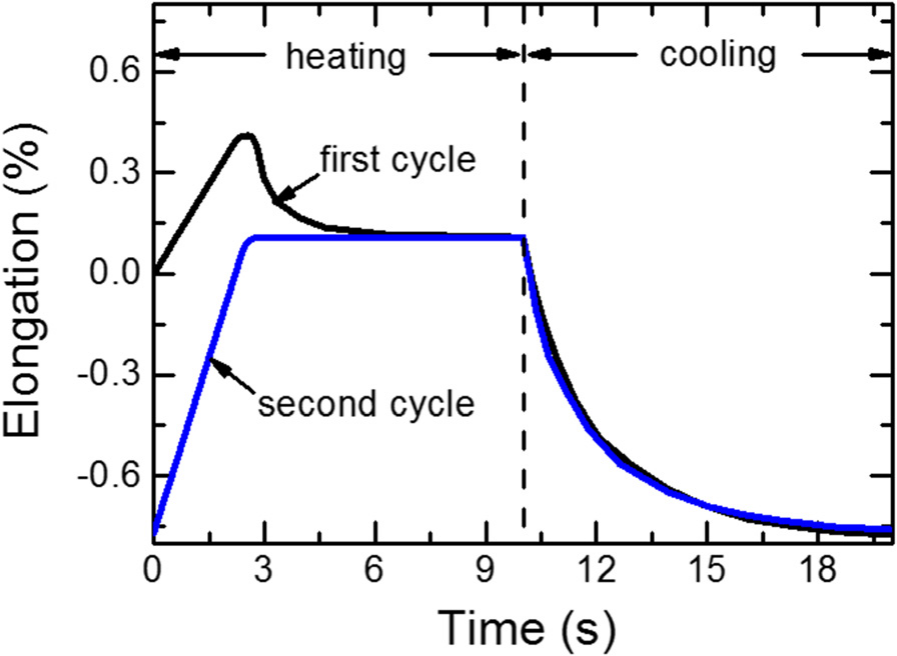
**Dependence of ribbon relative elongation on electric current heating time.** Current density *j*_*h*_ = 42 А/mm^2^. Heating time *t*_*h*_ = 10 s.

To understand what is happening with the ribbon during rapid heating, we have measured the relative elongation of the ribbon. Figure 4 shows the dependence of relative elongation on time for 2 cycles of heating and cooling. It is seen that after the current switch on, the ribbon extends as a result of thermal expansion. However, after 2.7 s, the length of the ribbon rapidly decreases, and after the power switch off and cooling to the initial temperature (20°C), the ribbon becomes shorter than it was in the initial state. This behavior occurs only during the first cycle of heating and cooling. The behavior of the relative elongation of the ribbon during the second cycle of heating-cooling is predictable: maximum thermal elongation of the ribbon (till the power is turned off) is observed after reaching the maximum temperature (after 3 s).

**Figure 5:**
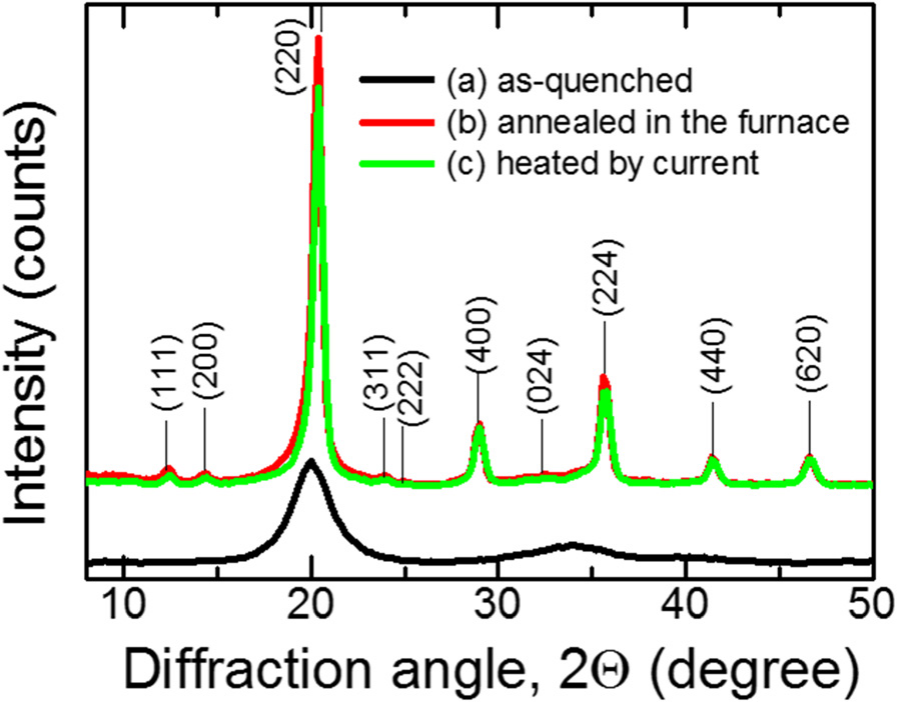
**X-ray diffraction patterns of the ribbons.** (Mo K_a_ radiation): (curve a) as-quenched amorphous ribbon, (curve b) after the annealing in a furnace (*T*_*a*_ = 550°C, *t*_*a*_ = 90 min), and (curve c) after the heating by electric current (*j*_*h*_ = 42 A/mm^2^, *t*_*h*_ = 3.7 s).

Thus, observing dilatometer effects, it was found that during the first cycle of heating-cooling, irreversible structural changes occur in the as-quenched amorphous ribbon that are related to the nanocrystallization process of the ribbon during its electric current rapid heating. This is supported by X-ray diffraction studies of the ribbon before and after annealing (Figure 5). Broadened reflections are observed in X-ray diffraction pattern that indicates the formation of large volume fractions DO_3_-type ordered nanocrystals of α-Fe(Si) solid solution.

It is known [5] that the low-temperature treatment of a similar amorphous alloy causes formation of Cu clusters, and the size of these clusters increases with annealing time increase from 5 to 60 min at *T*_*a*_ = 400°C. Those Cu clusters become nucleation centers for the formation of crystalline α-Fe phase. Contrary to the above treatment, during high-speed heating by electric current, Cu atoms do not have enough time to form relatively large clusters; this results in formation of increased number of α-Fe nanocrystals with decreased size: average crystal size of α-Fe in the amorphous matrix after annealing in furnace is 7 nm and after high-speed heating is 6 nm. The formation of smaller (as compared to the traditional heat treatment in a furnace) nanocrystals and their higher volume fraction qualitatively correlate with the data of the paper [16].

It is most likely that the reason of improving the magnetic characteristics of cores made from the ribbon heated by applying a certain density current, in particular, the reduction in core loss and significant increase of initial permeability *μ*_i_ (Figure 3), is the formation (by the time 3.7 s) of optimal volume fraction of nanocrystals with minimal size in the residual amorphous matrix.

It should be noted that the ribbon subjected to heating for less than 3.7 s is less fragile. This fact is in compliance with earlier results obtained in refs. [13,15] where the reduction of brittleness with a decrease of isothermal annealing time was observed. Increasing time of heating by electric current leads to noticeable increasing in the brittleness of the ribbon and deterioration of magnetic properties (see Figure 3, *t* > 3.7 s), which is likely due to the formation of larger nanocrystals and possible negative influence of surface oxidation.

Note that the values of the initial permeability *μ*_i_ of the current-heated ribbon are relatively low (less than 5,000) (Figure 3a). We supposed that this is related to the tension that appears in the ribbon after winding, especially when the inner diameter of the core is less than the critical diameter (*D* < *D*_c_) [17,21].

**Figure 6:**
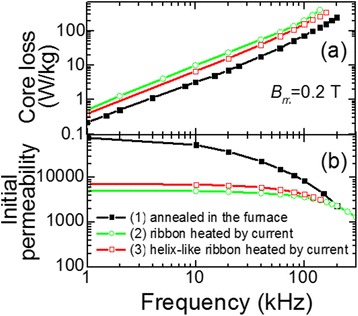
**Dependencies of core loss (a) and initial magnetic permeability (b) on frequency.** (1) Core annealed in furnace (*T*_*a*_ = 550°C, *t*_*a*_ = 90 min). (2) Core made of ribbon heated by electric current (*j*_*h*_ = 42 A/mm^2^, *t*_*h*_ = 3.7 s). (3) Core made of helix-like ribbon heated by electric current (*j*_*h*_ = 42 A/mm^2^, *t*_*h*_ = 3.7 s).

To check this assumption, we have compared the magnetic properties of three types of cores: (1) core made of initial ribbon and afterwards annealed in a furnace (550°C, 0.5 to 1.5 h), (2) core made of the current-heated ribbon, and (3) core made of the helix shape ribbon (the ribbon that was at first wound in tubular-like helix on a cylindrical holder and then heated by electric current; this ribbon retains its helix shape after cooling). Figure 6 shows the frequency dependencies of the initial permeability values *μ*_i_ (a) and core loss (b) for all three types of cores. It is seen that initial permeability values at 10 kHz, *μ*_i10_, for these cores are 54,000, 5,000, and 7,000, respectively. Thus, one can conclude that the tension in the third type of core is less than in the second type that leads to the *μ*_i10_ increase by 40%, which is still much lower than value *μ*_i10_ = 54,000 that is characteristic of the first type of core. Similarly, the core loss can also be reduced by decreasing the tension in the core (Figure 6).

Much lower permeability *μ*_і10_ in the cores made of the current-annealed ribbon as compared to the core annealed in a furnace indicates that, except for the mechanical tension, the ribbon is characterized by significant transverse magnetic anisotropy. The presence of this significant transverse anisotropy is also demonstrated by a reduction of remanence to saturation ratio *B*_*r*_/*B*_*s*_ (remanence induction *B*_*r*_ to saturation induction *B*_*s*_ ratio). Indeed, *B*_*r*_/*B*_*s*_ = 0.7 for core annealed in the furnace at *Т*_*а*_ = 550°С, *t*_*a*_ = 90 min, and *B*_*r*_/*B*_*s*_ = 0.43 for the core heated by electric current at *j*_*h*_ = 42 А/mm^2^ and *t*_*h*_ = 3.7 s. The possible cause of the additional magnetic anisotropy in amorphous ribbons (not related to the tensile stress) that is induced during nanocrystallization at rapid heating is, in our opinion, the influence of the surrounding atmosphere, e.g., anisotropic oxidation and hydrogenation of the ribbon and consequent anisotropic crystallization of the ribbon surfaces [22].

It should be noted that the ribbon heated by electric current is characterized by low saturation magnetostriction, so the core is insensitive to deformations - this follows from the fact that the initial permeability remains unchanged under strong deformation of the core up to ½ of its initial diameter. Different behavior is observed for the core annealed in the furnace: deformation of the core causes decreasing of permeability by more than seven times at the frequency of 10 kHz and three times at the frequency of 100 kHz.

### Rapid heating of the ribbon by electric current under tensile stress

**Figure 7:**
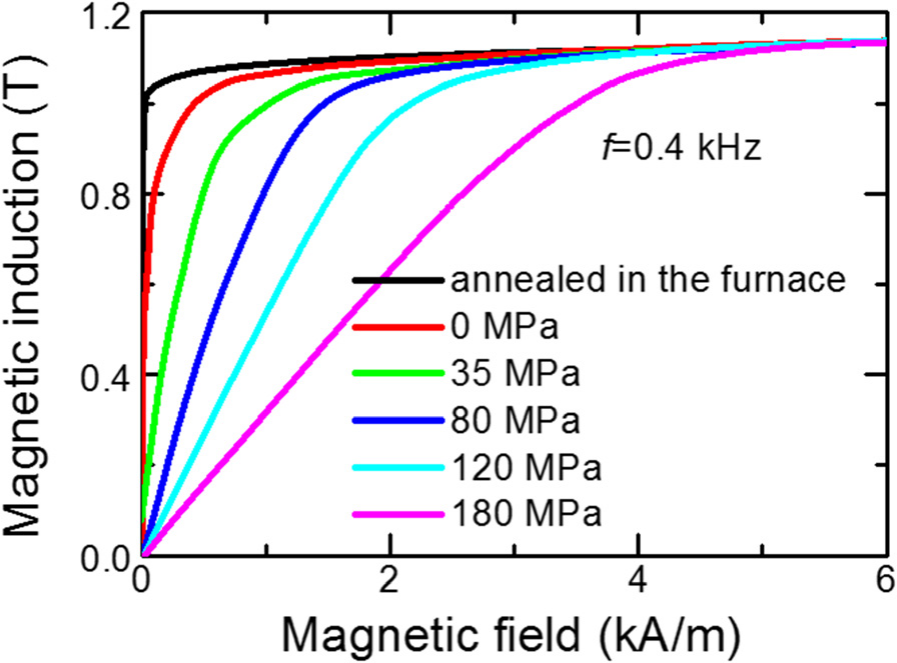
**Remagnetization loops of the cores made of ribbon.** Heated by electric current (*j*_*h*_ = 42 A/mm2, *t*_*h*_ =3.7 s) under tensile stress in the range 0 ≤ *σ* ≤ 180 MPa and annealed in a furnace at *T*_*a*_ = 550°C, *t*_*a*_ = 90 min.

Amorphous ribbon has been crystallized during rapid heating by electric current at simultaneous applying of tensile stress *σ* along the ribbon axis. The heating under tensile stress causes strong transverse anisotropy of the ribbon related to creep [10]. It was shown in [11,12,16] that the anisotropy linearly increases with increasing of tensile stress *σ*. It can be seen from Figure 7 that increasing of tensile stress leads to increasing of a magnetic field required to achieve saturation induction *B*_*s*_.

**Figure 8:**
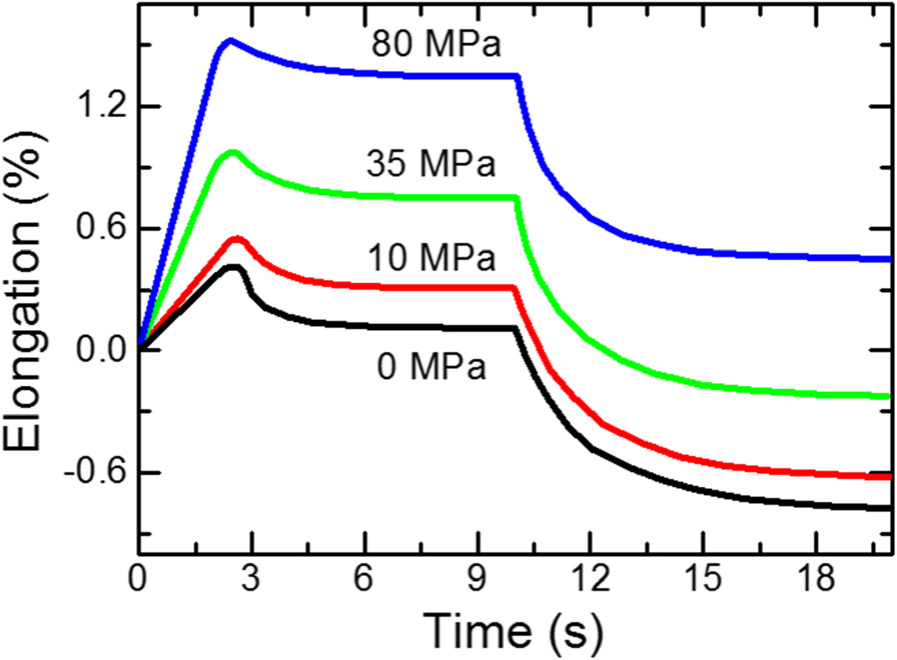
**Dependence of relative elongation of ribbon on time.** *j*_*h*_ = 42 A/mm^2^, *t*_*h*_ = 10 s.

Figure 8 presents the dependence of the relative ribbon elongation on time. Increase of the tensile stress from 0 to 80 MPa leads to the final elongation increase from −0.77% to 0.45%. It was shown [15] that final elongation linearly depends on load but with considerably higher values of elongation that probably are due to the different conditions of annealing of the ribbon. It was also found that the magnitude of the applied tensile stress does not affect the position and height of the diffraction peaks, i.e., the diffraction patterns obtained from the specimen heated with and without tensile stress are the same. This confirms the conclusion [10] that the main contribution to the induced magnetic anisotropy originates from the magnetoelastic anisotropy of Fe-enriched grains due tensile back stresses exerted by inelastically deformed amorphous matrix.

**Figure 9:**
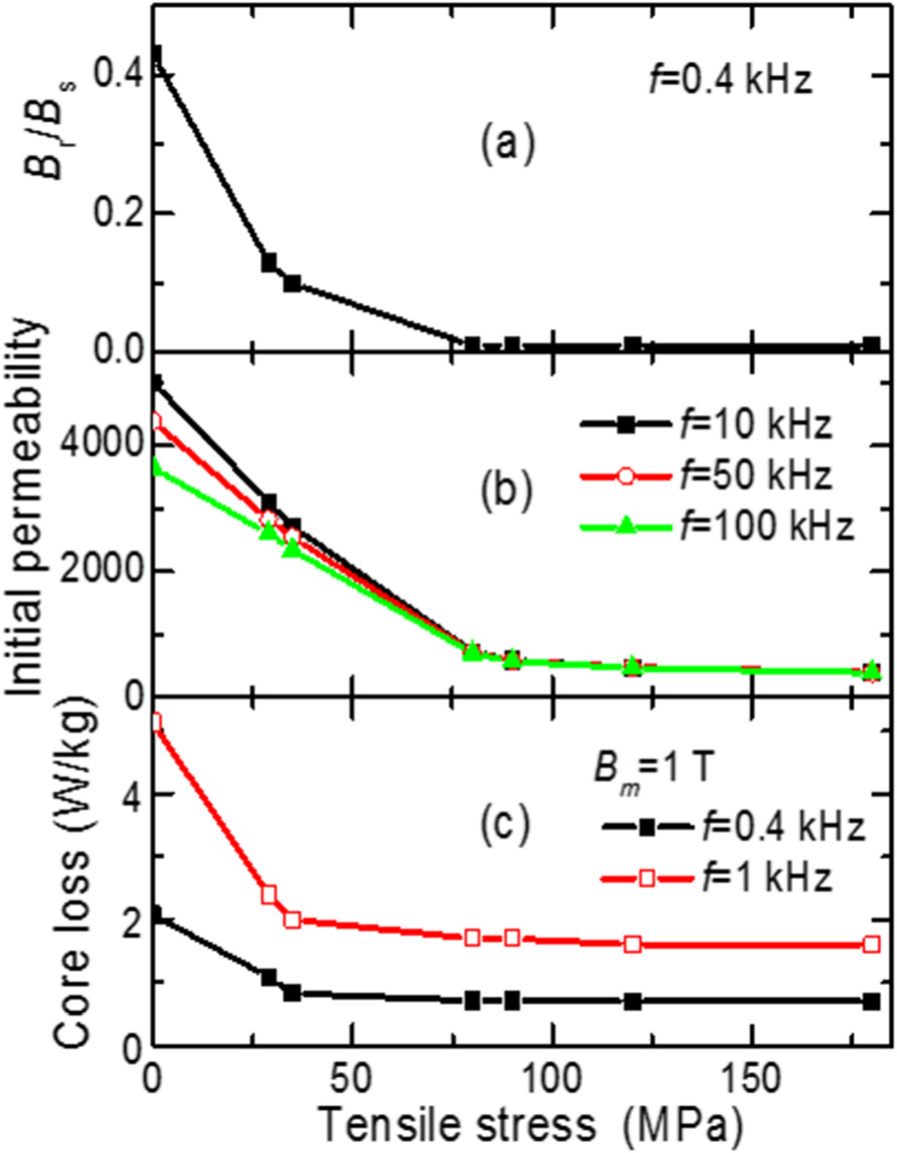
**Dependence of magnetic properties on tensile stress.** *j*_*h*_ = 42 A/mm^2^, *t*_*h*_ = 3.7 s: **(a)** remanence to saturation ratio *B*_*r*_/*B*_*s*_ vs. tensile stress, **(b)** initial magnetic permeability *μ*_і_ vs. tensile stress, and **(c)** core loss vs. tensile stress.

The tensile stress increase from 0 to 180 MPa results in the decrease of the following values: remanence to saturation ratio from 0.43 to 0.01 (Figure 9a), initial magnetic permeability *μ*_i10_ to 400 (Figure 9b), and core loss by almost three times (2.08 → 0.7 W/kg) at low frequency 400 Hz and 1.0 T maximal induction (Figure 9c). Reduced permeability is consistent with the results obtained in [15,16] while the resulting reduction of the loss by heating under stress is inconsistent with the results obtained in [8,12], where it was shown that an increase in transverse anisotropy led to an increase of both relative loss per cycle [8] and coercitivity [12]. Perhaps this difference is associated with a different way of ribbon heating.

**Figure 10:**
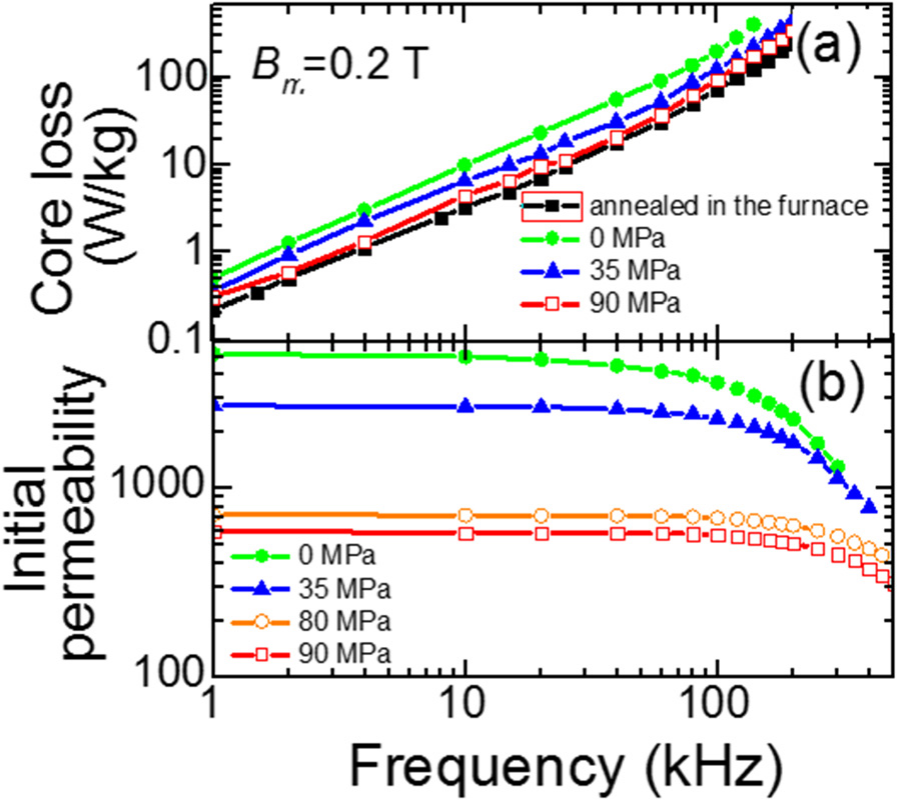
**Dependencies of core loss (a) and initial magnetic permeability (b) on frequency.** Core annealed in furnace (*T*_*a*_ = 550°C, *t*_*a*_ = 90 min). Cores made of ribbon heated by electric current (*j*_*h*_ = 42 A/mm^2^, *t*_*h*_ = 3.7 s) under the tensile stress in the range 0 ≤ *σ* ≤ 90 MPa.

Figure 10a shows the dependences of core loss on the frequency in the range 1 to 300 kHz measured at maximal induction 0.2 T. The values of the applied stress varied in the range 0 to 90 MPa. It is seen that the increase of the tensile stress leads to the decrease of core loss in the whole frequency range. At 90 MPa, the loss is comparable with the one observed for the core made of the ribbon annealed in the furnace.

Investigation of the frequency dependence of initial magnetic permeability showed that the increase of the tensile stress from 0 to 90 MPa during heating improves the frequency stability of initial permeability of cores (Figure 10b). These results correlate with the papers [13,17] that report the frequency dependence of initial relative permeability of the cores made from the ribbon heated under even higher tensile stresses 100 and 200 MPa.

### Comparison of the magnetic properties of the cut core with the core made of the ribbon rapid heated under tensile stress

**Figure 11:**
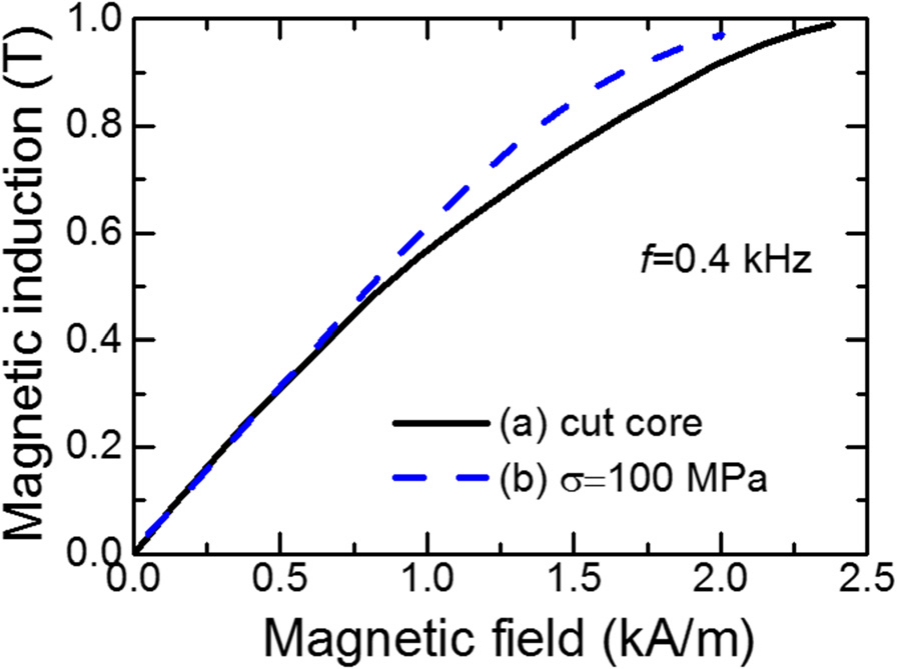
**Remagnetization loops of cores.** (Curve a) cut core with non-magnetic gap 0.045 mm (*T*_*a*_ = 550°C, *t*_*a*_ = 90 min) and (curve b) core made of ribbon heated by electric current (*j*_*h*_ = 42 A/mm^2^, *t*_*h*_ = 3.7 s) under tensile stress 100 MPa.

In the present section, we compare basic magnetic properties of cut core made of nanocrystalline Fe_73_B_7_Si_16_Nb_3_Cu_1_ alloy (inner/outer diameter 30/42 after the annealing in the furnace at *T*_*a*_ = 550°C for 90 min) and new core made of the ribbon heated by current under tensile stress at 100 MPa with the same dimension. The gap between the two halves of the cut core was adjusted to obtain the initial permeability *μ*_i10_ = 500 equal to the permeability of the core made of the rapid-heated ribbon (under tensile stress at 100 MPa). Remagnetization loops of new cores and cut core are presented in Figure 11. It is seen that the cut core loop in the induction range of 0 T ≤ *B* ≤ 0.8 T has a rounded shape while the new core, made of the ribbon with induced transverse anisotropy, is characterized by much higher linearity of remagnetization loop, which means independence of the core effective permeability on magnetic field.

**Figure 12:**
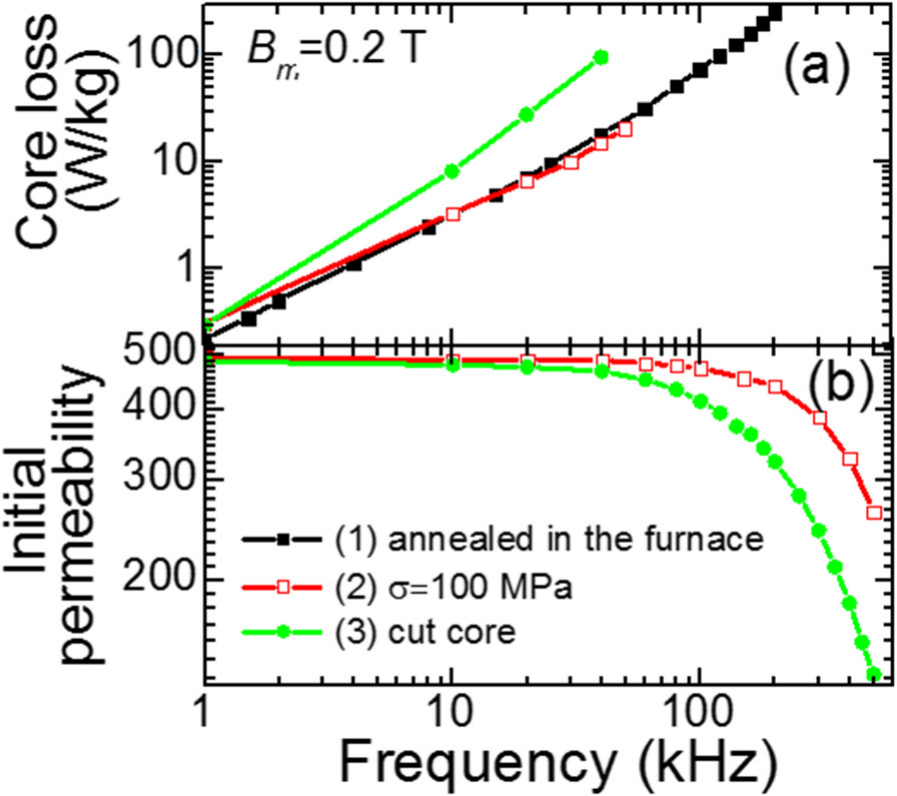
**Dependencies of core loss (a) and initial magnetic permeability (b) on frequency.** (1) Core annealed in furnace (*T*_*a*_ = 550°C, *t*_*a*_ = 90 min). (2) Core made of ribbon heated by electric current (*j*_*h*_ = 42 A/mm^2^, *t*_*h*_ = 3.7 s) under tensile stress 100 MPa. (3) Cut core with non-magnetic gap 0.045 mm (*T*_*a*_ = 550°C, *t*_*a*_ = 90 min).

The dependence of the core loss on the frequency *f* for the cut and new gapless cores is shown in Figure 12a. It is seen that at the frequencies above 1 kHz, the core made of the ribbon with transverse anisotropy (heated by current under tensile stress) show a significant advantage in loss as compared to cut core.

Investigation of the frequency dependence of initial magnetic permeability showed that the new core had higher frequency stability than the cut core (Figure 12b). A new toroidal core had constant initial permeability up to 100 kHz. Higher frequency stability and high linearity of loops are significant advantages for producing filter inductors with high impedance stability throughout the range of operating currents.

## Conclusions

1. The optimal rapid electric heating modes (*j*_*h*_ = 42 А/mm^2^, *t*_*h*_ = 3.7 s) of the amorphous ribbon allow to reach the maximum improvement of magnetic characteristics of cores made of the heated ribbon: initial magnetic permeability increase and core loss decrease. It is shown that during rapid annealing, the magnetic anisotropy is induced in the ribbon. It is, probably, caused by the anisotropic oxidation of the ribbon by the consequent anisotropic crystallization of the ribbon surfaces.
2. The tensile stress increase from 0 to 180 MPa at rapid electric current heating results in the decrease of initial magnetic permeability (*μ*_10_ = 5,000 → 400), remanence to saturation ratio (from 0.43 to 0.01), and core loss at frequencies from 10 to 300 kHz. In particular, core loss decreases by three times (2.08 → 0.7 W/kg) at low frequency 400 Hz, maximum magnetic induction 1.0 Т. The tensile stress increase from 0 to 90 MPa during the heating of ribbons improves frequency stability of initial magnetic permeability of cores made of these ribbons.
3. Principal magnetic properties of the cores made of ribbons with transverse anisotropy (heated by electric current under tensile stress) are better than those of cores with non-magnetic gap. First of all, core loss is considerably lower at frequencies above 1 kHz; remagnetization loop is characterized by higher linearity in the range of 0 Т ≤ *B* ≤ 0.8 T and frequency stability of initial magnetic permeability increases.

The obtained characteristics are advantageous for applications of (Fe_73_B_7_Si_16_Nb_3_Cu_1_)-based magnetic cores in power reactors and linear chokes of filters of switch-mode power supplies.
